# Heat transfer analysis of underground U-type heat exchanger of ground source heat pump system

**DOI:** 10.1186/s40064-016-3548-8

**Published:** 2016-10-24

**Authors:** Guihong Pei, Liyin Zhang

**Affiliations:** School of Civil Engineering and Architecture, Southwest Petroleum University, Chengdu, 610500 China

**Keywords:** U-type ground tube, Numerical simulation, Heat transfer rate, Thermal interference

## Abstract

**Background:**

Ground source heat pumps is a building energy conservation technique. The underground buried pipe heat exchanging system of a ground source heat pump (GSHP) is the basis for the normal operation of an entire heat pump system.

**Methods:**

Computational-fluid-dynamics (CFD) numerical simulation software, ANSYS-FLUENT17.0 have been performed the calculations under the working conditions of a continuous and intermittent operation over 7 days on a GSHP with a single-well, single-U and double-U heat exchanger and the impact of single-U and double-U buried heat pipes on the surrounding rock-soil temperature field and the impact of intermittent operation and continuous operation on the outlet water temperature.

**Conclusions:**

The influence on the rock-soil temperature is approximately 13 % higher for the double-U heat exchanger than that of the single-U heat exchanger. The extracted energy of the intermittent operation is 36.44 kw·h higher than that of the continuous mode, although the running time is lower than that of continuous mode, over the course of 7 days. The thermal interference loss and quantity of heat exchanged for unit well depths at steady-state condition of 2.5 De, 3 De, 4 De, 4.5 De, 5 De, 5.5 De and 6 De of sidetube spacing are detailed in this work. The simulation results of seven working conditions are compared. It is recommended that the side-tube spacing of double-U underground pipes shall be greater than or equal to five times of outer diameter (borehole diameter: 180 mm).

## Background

As a building energy conservation technique, ground source heat pumps (GSHP) have attracted considerable attention on the basis of rising energy prices and urgent environmental pressure caused by excessive energy consumption. Bhutta et al. (2012) reviewed CFD techniques and concluded that they are good tools to simulate heat exchanger design. The geothermal heat exchangers (GHEs) are the basis for the normal operation of heat pump systems, and the thermal characteristics significantly depend on the rock-soil type and the longitudinal temperature distribution. In addition, heat transfer with the surrounding rock-soil is a complicated and unstable process. There has been significant research into the heat transfer phenomena of GHEs which has been which include experimentation, numerical simulation and theoretical analysis models (Bouhacina et al. 2015; Hu et al. 2013; Nam et al. 2008). Katsura (2008) proposed a method to calculate the rock-soil temperature under a pipe-group heat extraction condition by means of a temperature superposition based on linear heat source theory. Shang (2011) predicted the rock-soil temperature variation between the operation and recovery period of GSHPs by establishing a 3D model of a single-well, single-U heat exchanger combined with multi-aperture theory. The results indicated that the soil properties have a greater influence on the soil temperature recovery than environmental factors did. Choi (2011) applied a CFD numerical simulation method to analyzing the influential factors which contains shank spacing, borehole depth, flow velocity and the differential temperature of the inlet/outlet on the heat transfer rate of the GSHP. The results showed that the borehole depth was found to be the most significant factor affecting the system performance. Additionally, the impact of the saturated soil on the mean heat exchange rate was higher than in unsaturated soil, at 40 %. However, to facilitate the research, it is assumed that the soil is homogeneous.

Generally, the drilling diameter is 100~300 mm (Zhao and Dai 2007). Among all GSHP vertical ground heat exchangers, u-tubes, annular tubes and single tubes are most commonly used. The U-tube is most common due to its simple construction, good heat exchanger performance, high bearing, less tube joints, and unlikely leakage. The U-type side tube spacing is small due to restraints imposed on the well drilling diameter. Due to the existence of temperature differences, heat conduction will occur directly between the two side-tubes, interfering with the ability of the soil to function as an effective heat sink. Thermal interference seriously influences the underground heat exchange of U-type tubes, decreasing the quantity of heat rejection for unit well depth by 20–40 %. More seriously, it will lead to a malfunction of the heat pump refrigerant-cycle system, which can stop operation. It was found that increasing the pipe spacing, applying high thermal conductivity backfill soil can enhance the performace of GSHP systems (Dehkordi and Schincariol 2014). Shen (2007) uses a finite unit method to perform quantitative analyses on the hystereses caused by thermal interference. Carli (2010) analyzed the thermal interference in the drill holes by calculating the heat resistance, which makes uses an electrical analogy with lumped capacitances. These thermal resistances were used to solve for the heat transfer in an unsteady state. For the vertical double U-type ground tube, although the quantity of heat exchange for a unit well depth is larger than the single U-type tube, for the same drilling area, thermal interference is more likely to happen because the temperature of several side-tubes is different. The rock-soil’s heat equilibrium temperature not only affects the underground rock-soil heat transfer rate, but is also associated with normal operation and economy of heat pump systems. An ideal temperature range ensures efficient running of the system. In addition, appropriate side-tube spacing not can enhance the heat exchanger efficiency but also can reduce the required drilling diameter. Therefore, this paper establishes a three-dimensional heat transfer model for a single-well, double-U buried tube based on the rock-soil thermophysical properties. It also accounts for vertical temperature stratification according to underground heat transfer test experiment conditions, and carried out comparison of simulation results for soil temperature and U-tube water temperature are compared for single U-type buried pipes and a double-U buried pipes for continuous operation over 7 days. After that, the paper compares the outlet water temperature changes during the time of continuous operation and intermittent operation for a double-U heat exchanger. Eventually, the double-U buried pipe model was established of different side-tube center distance. Thermal interference arising from temperature distribution variation for different side-tubes was also analyzed.

## Methods

### Governing equation

The process of fluid flow and heat conduction within U-type GHEs follows the law of conservation laws of mass, momentum and energy. The mathematical description of these laws is the controlling equation of the process. Since the *Re* is 18,116, we use standard *k*-*ε* model to simulate the turbulent flow, which has widely applicability, robustness, and saves computation time. The general governing equation (Tu et al. 2009) is as follows:

1 $$\frac{\partial \phi }{\partial t} + \frac{{\partial \left( {u\phi } \right)}}{\partial x} + \frac{{\partial \left( {v\phi } \right)}}{\partial y} + \frac{{\partial \left( {w\phi } \right)}}{\partial z} = \frac{\partial }{\partial x}\left( {Gamma \frac{\partial \phi }{\partial x}} \right) + \frac{\partial }{\partial y}\left( {\Gamma \frac{\partial \phi }{\partial y}} \right) + \frac{\partial }{\partial z}\left( {\Gamma \frac{\partial \phi }{\partial z}} \right) + S_{\phi }$$

Continuity part

$$\phi = 1;\Gamma = 0;S_{\phi } = 0$$

Momentum part

$$\phi = u,v,w;\Gamma = \nu + \nu_{T} ;S_{\phi } = - \frac{1}{\rho }\frac{\partial p}{\partial x}S_{u}^{'} , - \frac{1}{\rho }\frac{\partial p}{\partial y}S_{v}^{'} , - \frac{1}{\rho }\frac{\partial p}{\partial z}S_{w}^{'}$$

Energy part

$$\phi = T;\Gamma = \frac{\nu }{\Pr } + \frac{{\nu_{T} }}{{\Pr_{T} }};S_{\phi } = S_{T} ;$$

Turbulent kinetic energy *k* and turbulent energy dissipationεpart

$$\phi = k,\varepsilon ;\Gamma = \frac{{\nu_{T} }}{{\sigma_{k} }},\frac{{\nu_{T} }}{{\sigma_{\varepsilon } }};S_{\phi } = P - D,\frac{\varepsilon }{k}(c_{\varepsilon 1} P - c_{\varepsilon 2} D)$$

where *u*, *v*, *w*, stands for the velocity of *x*, *y*, *z* dimension, respectively. *ν* means kinematic viscosity. *t*, *T*, *ρ* and *p* stands for time, temperature, density and pressure, respectively; Pr is Prandtl number, which means the ratio of molecular momentum diffusivity and molecular thermal diffusivity. Subscript *T* stands for turbulent flow, *P* means the term of turbulent kinetic energy production, and *D* means the term of turbulent energy dissipation. Constants for the turbulent model are observed as below (Launder and Spalding 1974):

$$\upsigma_{\text{k}}=1.0, \upsigma_{\varepsilon}=1.3, {\text{c}}_{\varepsilon1}=1.44, {\text{c}}_{\varepsilon2}=1.92$$

#### The geometric model

**Fig. 1:**
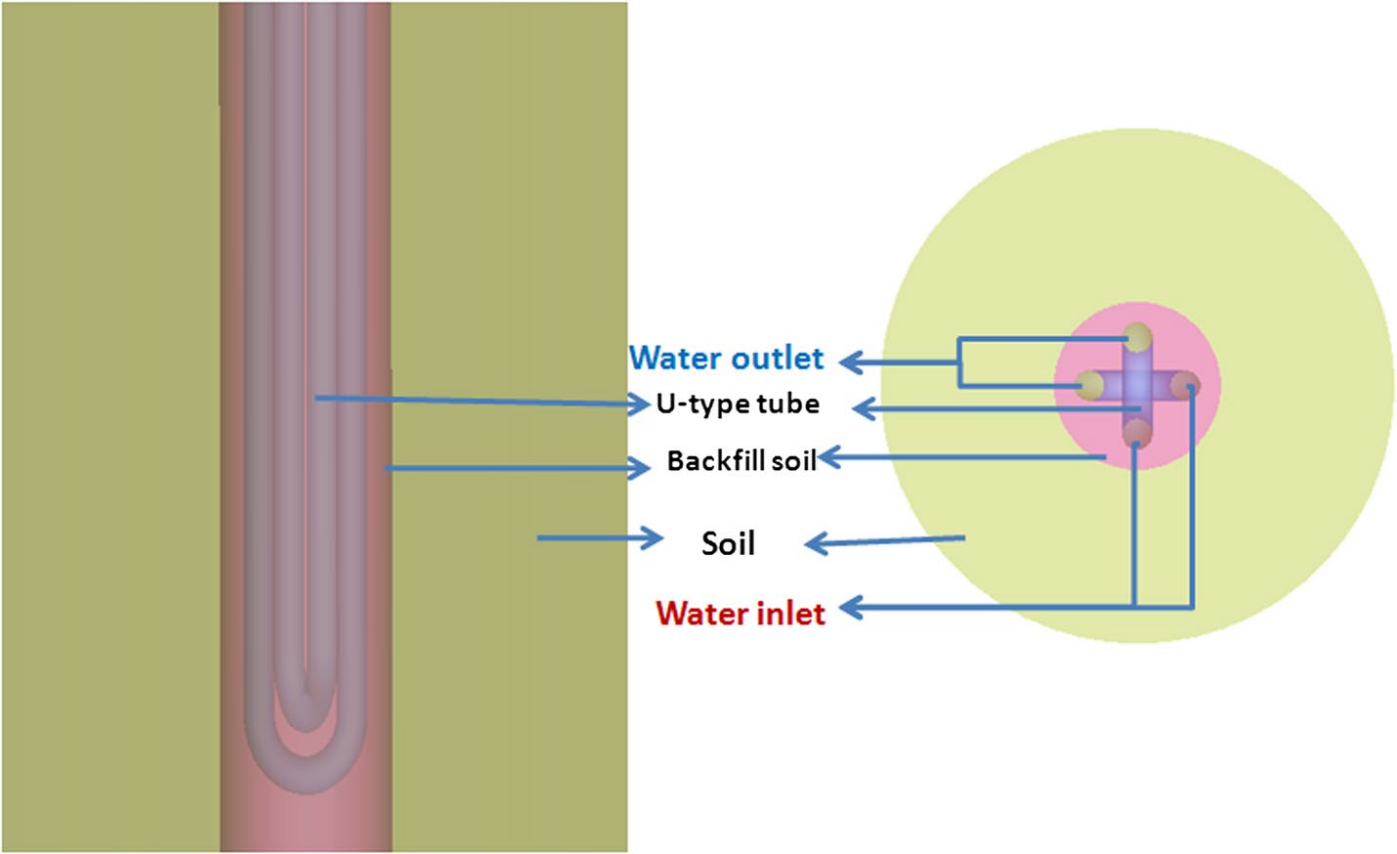
Schematic diagram of geometric model

**Fig. 2:**
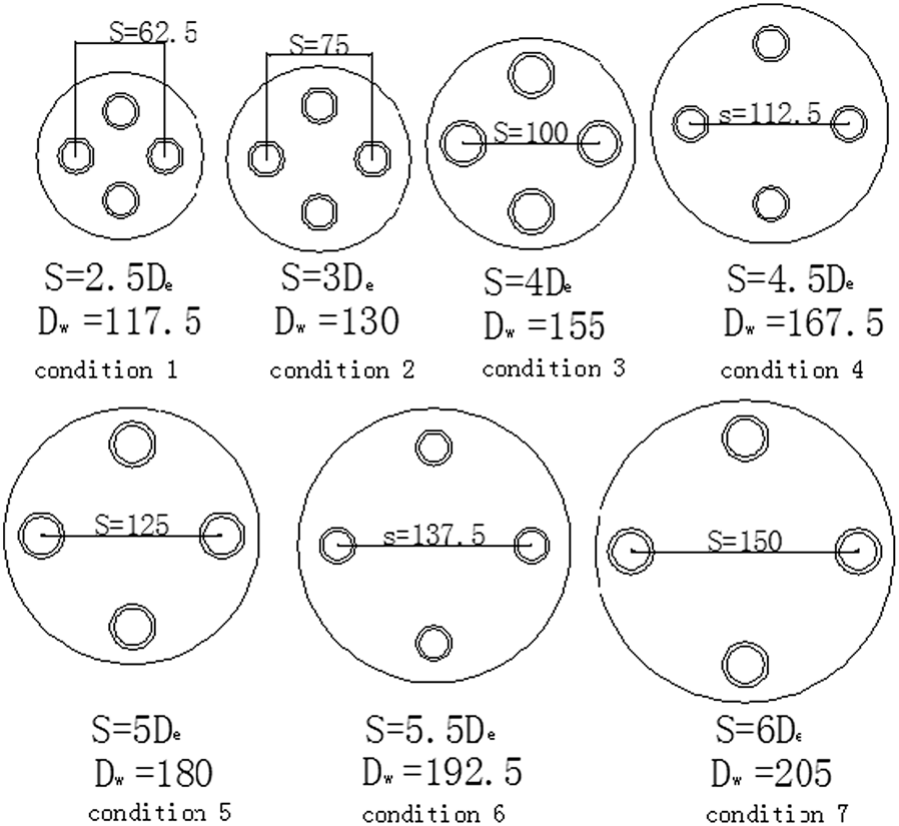
Schematic diagram of the cross section with different center distance and well

ANSYS-ICEM CFD (ANSYS I. C. 17.0 2016) pre-processing software to build the geometric model of the single-U and double-U ground heat exchanger and mesh the model. The simulated U-type tube is a DN25 HDPE tube, with the standard specification, (Sun et al. 2000) which has an outer diameter 25 mm, inner diameter of 20.4 mm, wall thickness of 2.3 mm, and a tube surface roughness of 0.01 mm. According to the Prandtl–Schlichting equation, the thickness of the viscous sublayer is 0.29 mm which indicates that the turbulence is in the hydraulic smooth wall region. The side-tube spacing is 75 mm with drilling a diameter of 130 mm. The ground far boundary semi-diameter is 2.5 m, and the distance from the bottom surface to the pipe’s elbow is 1 m. The embedded depth of the tube is 80 m and the tube’s center distance S equal 3 D_e_. A schematic diagram of geometric model is shown in Fig. 1. Based on the basic model, we build the geometric model of a single well double-U tube with S/D_e_ = 2.5, 3, 4, 4.5, 5, 5.5, and 6. The cross section is shown in Fig. 2, and the units are mm.

### Numerical calculation

ANSYS FLUENT 17.0 (2016) software is used to calculate the current numerical investigation. A finite volume discretization is used in approximating the governing equations. A double-precision and pressure-based solver is used in the numerical computation. A non-slip boundary condition is adopted on pipe surface. The SIMPLE algorithm is used for pressure–velocity coupling. A first-order upwind scheme is adopted to the discretization of all terms. The computation can be considered as converged when the normalized residuals for mass, momentum and energy equations are less than 10^−6^, 10^−6^, 10^−8^, respectively. The final results are the dynamic simulation values with a time-step size of 30 s for 7 days of continuous/intermittent operation. The intermittent operation mode process is within the specific time steps where the inlet velocity and flow mode will go through periodical changes between 0.65 m/s, standard k-ε mode and 0.0 m/s, laminar mode. The inlet temperature is set as 303 K, during the shutoff operation. The following assumptions can be implemented:

1. Ignore the influence of surface temperature fluctuations on the ground temperature.
2. Ignore the influence of the water migration in the soil.
3. Ignore the thermal contact resistance between U-type tube to backfill material, backfill material to well wall, and well wall to the soil.

#### Grid generation

**Fig. 3:**
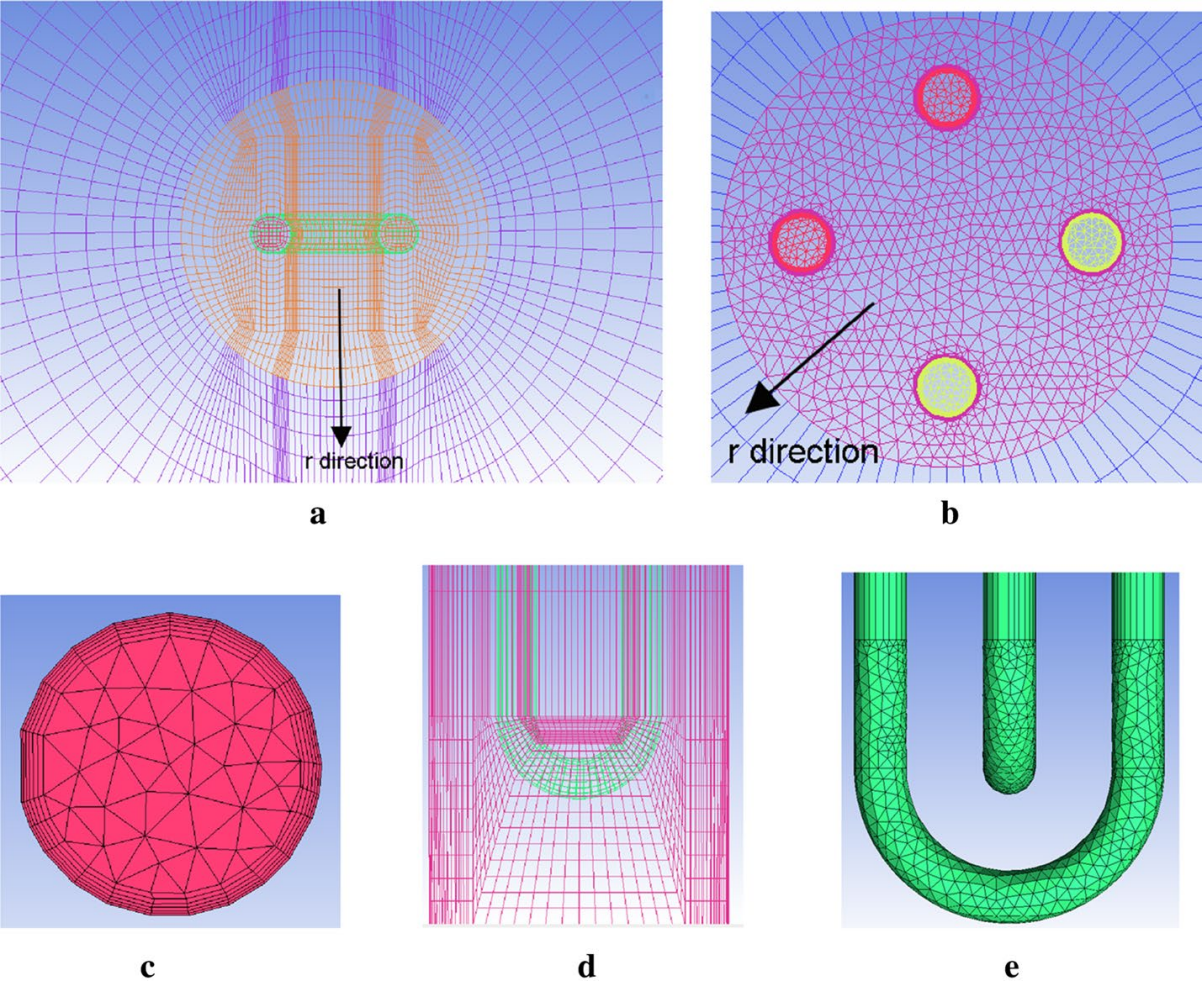
The mesh of cross-section (**a**–**b**), inlet of double-U (**c**), elbow (**d**–**e**)

The single-U model in this work uses structured meshing while the double-U models all use a combination of structured and non-structured meshing. In the simulation, structured meshing and non-structured meshing can provide the same precision (Shevchuk et al. 2011). To meet the requirement of the turbulence model, we use a growing layer ratio of 1.2 to satisfy the desired y+ value, dividing the prism into six layers where the thickness of first layer is 0.1 mm in the radial direction and is adjacent to the interface of the water domain. Because the entire model is a spindly structure, a mesh size of 100 mm is adopted in the longitudinal direction. The mesh size in the elbow of a U-type tube is small approximately 2 mm, due to centrifugal force. The meshes in the soil part gradually sparse and their minimum size is 20 mm in the radial direction. Various mesh elements (approximately 25 million, 33 million and 40 million in single-U; approximately 30 million, 45 million, and 50 million in double-U) are generated to pass a mesh independence test. The relative deviations of the average outlet temperature on both U-type tubes between the last two sets of meshes are within 2 %. To reduce the quantity of mesh, the final number of cells for the single-U and double-U type GHEs systems are 3297878 and 4522149, respectively. Pictures of the mesh are shown in Fig. 3.

#### Boundary conditions and material’s physical property parameters

**Fig. 4:**
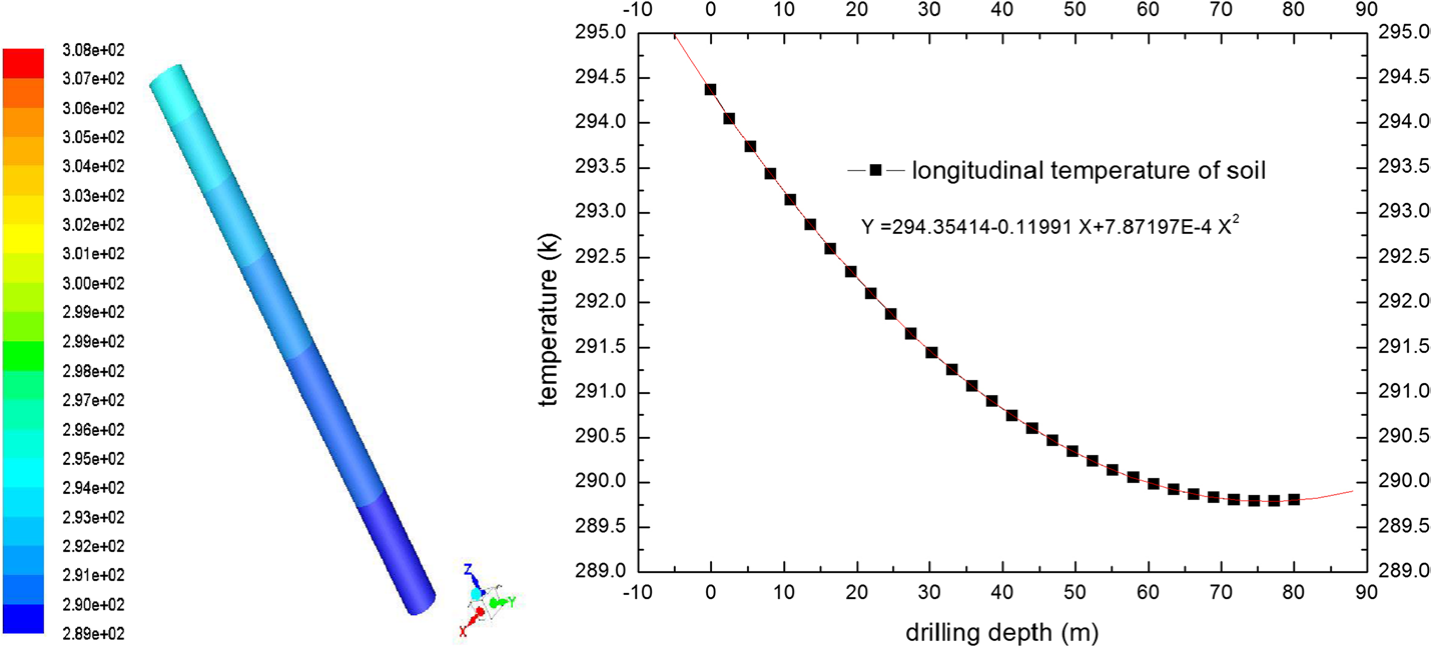
Distant surface and initial temperature of soil from *upper* to *bottom*

The computational domain material physical parameters are shown in Table 1. Boundary conditions are shown in Table 2. The initial temperature of all domains and soil boundary wall temperatures varies according to a quadratic function formula based on a numerical fitting of the experiment. These were compiled by INIT macro and PROFILE macro of UDF programs. Figure 4 shows soil temperature changes.

**Table 1:** Material’s physical property parameter

	ρ [kg/m^3^]	c_p_ [J/kg.k]	λ [W/m.k]	η [Pa.s]
Water	993.9	4147	0.6265	7.275e−04
HDPE pipe	950	2300	0.45	
Backfill soil	1900	900	2.2	
Rock-soil	2530	840	2.58

**Table 2:** Boundary conditions

	Boundary type	Velocity (m/s)	Temperature (K)
*Fluid part*			
Inlet	VELCITY_INLET	0.65	308
Outlet	OUTFLOW		
*Solid part*			
Distant surface	Gradually reduce from upper to bottom		
Borehole wall	Coupled wall		
Pipe wall	Coupled wall		289
Bottom surface	Constant temperature		
Top	Heat insulation

## Results and discussion

### Simulation verification

**Fig. 5:**
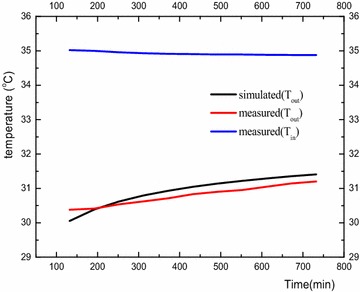
Comparison of outlet temperature between experiment and simulation

Figure 5 shows the test model of the working condition 2 (S = 3De). When operating continuously for 12 h, the temperature at the outlet of the U-type pipe changes with time. Table 3 is the comparison between the simulation value and the measured value. The definition of heat transfer for a unit well depth of a U-type heat exchanger as follows:

2 $${\text{q}}_{l} = \frac{{G \cdot c_{p} \cdot \left( {T_{in} - T_{out} } \right)}}{H}$$

**Table 3:** The comparison between simulation value and measured value

	Mass flow rate [kg/s]	T_in_ [°C]	T_out_ [°C]	ΔT [°C]	Q [KW]	q_l_ [w/m]
Experimental value	0.563	34.91	31.2	3.71	8.718	108.975
Simulation value	0.563	35	31.5	3.5	8.224	102.8

G is the mass flow rate in kg/s. c_p_ is specific heat in J/kg/K; T_in_ is the temperature of water inlet in K. T_out_ is the temperature of water outlet in K. H is the drilling depth in m.

As shown in Fig. 5, the outlet temperature is changing with time in agreement with the experimental data from (Zhang et al. 2009). From Table 3 we can see the simulation value of u-tube heat transfer at a unit well depth is 102.8 w/m, and the measured value is 108.975 w/m, which is a fractional error of 6.175 %. Thus the simulation results can be observed as valid.

### The temperature variation of water along the pipe and rock-soil

**Fig. 6:**
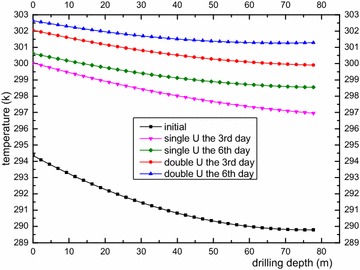
Longitudinal temperature change of rock-soil at r = 0.078 m

**Fig. 7:**
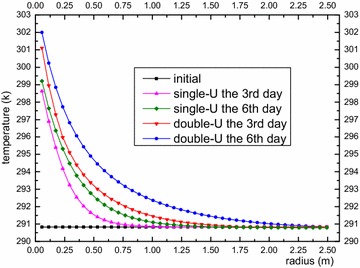
Radial temperature change of rock-soil at Z = −40 m

**Fig. 8:**
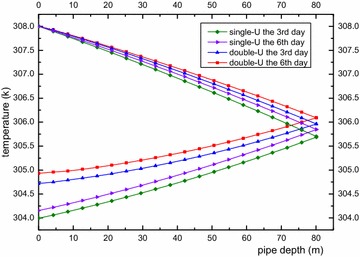
Average temperature variation within U-type pipe

In this section, we analyzed the impact of a single-U heat exchanger and double-U heat exchanger on the surrounding rock-soil temperature and fluid temperature under a continuous operation mode and an intermittent operation mode. Figures 6 and 7 show rock-soil temperature changes under the condition of a single-U and double-U buried pipe after running for 3 and 6 days at r = 0.078 m along the depth direction and z = −40 m along the radial direction. Figure 6 shows that the single-U heat exchanger raises temperature of rock-soil by 6.91 K and 8.0 K on average at the edge of the borehole and an average change of 38 and 44 % was observed relative to the initial average temperature (291.31 K). Furthermore, the double-U heat exchanger makes the temperature of the rock-soil rise by 9.39 and 10.38 K on average at the edge of the borehole. The average change rates have reached 51.7 and 57 % compared with initial average temperature. From above we can see that temperature increase rate of the rock-soil gradually decreases as the operation time increases. The impact of the double-U heat exchanger on the initial temperature of the rock-soil is approximately 13 % higher than that of the single-U heat exchanger. From Fig. 7 it can observed that the temperature of the rock-soil from the far boundary of the drilling location presents an increasing inverse proportional function. The influence of the double-U buried pipe radius is twice that of the single-U tube. Consequently, it is suggested that the drilling spacing of the single-U buried pipe should be reduced relative to the double-U buried pipe. Adopting a mixed pipe laying form, the double-U buried pipes lay on the outermost layer. Figure 8 indicates the serious heat short-circuiting of the double-U buried pipe. The decreasing outlet pipe water temperature is obviously higher than that of single-U buried pipe. The mean temperature difference between the inlet and outlet of the single-U buried pipe at the 3rd and 6th day are 4.01 and 3.85 K. Consequently, the rates of heat transfer are 9.423 and 9.047 kw and that of double-U buried pipe are 3.28 and 3.068 K. The heat transfer rates are 7.71 and 7.21 kw, respectively.

**Fig. 9:**
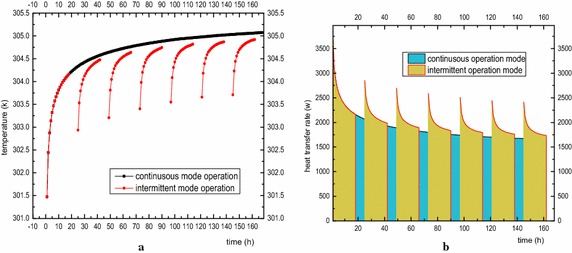
**a** Outlet fluid temperature variation and **b** heat transfer rate at continuous/intermittent operation mode

**Fig. 10:**
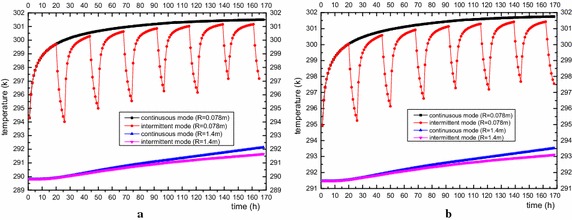
Rock-soil temperature variation at Z = −70 m (**a**) and Z = −30 m (**b**)

**Fig. 11:**
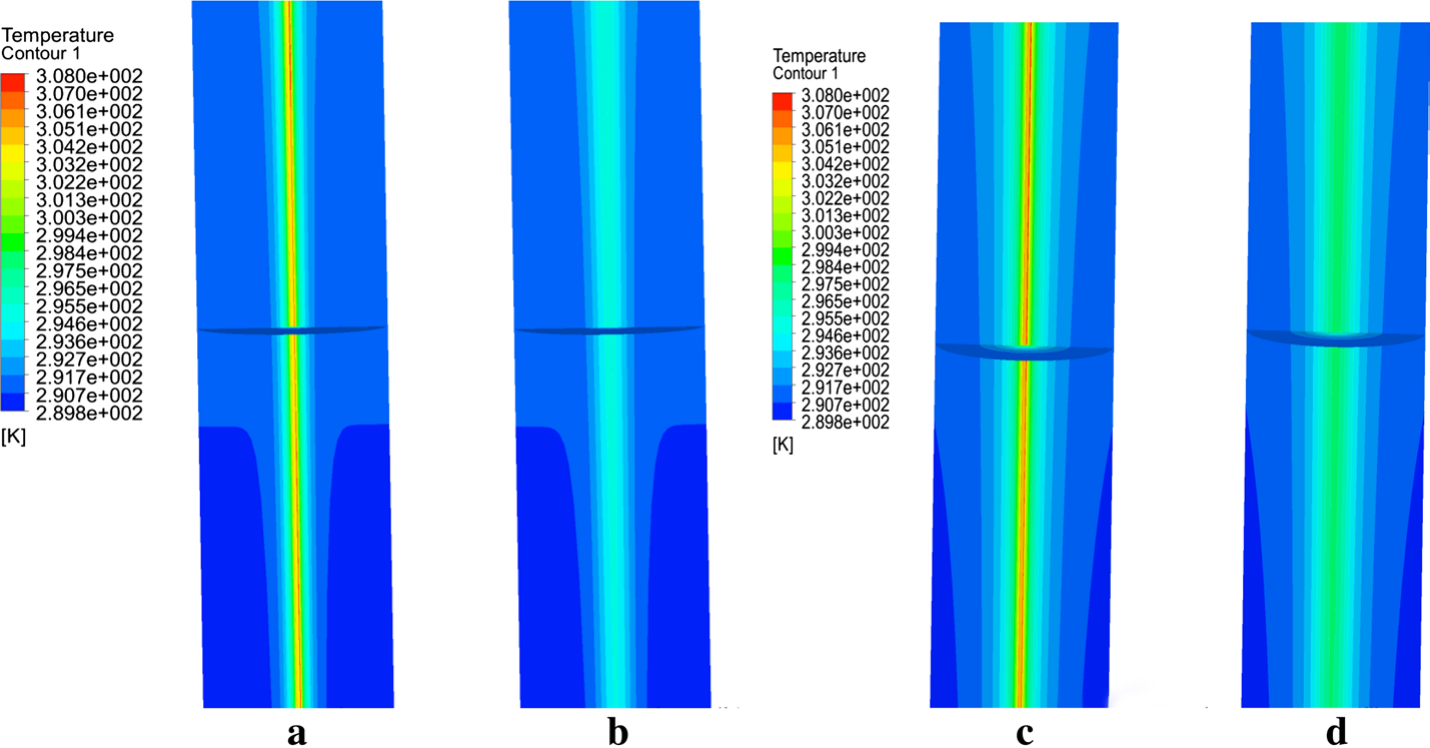
The temperature field in the longitudinal direction running at t = 18 h (**a**) and t = 138 h (**c**); out of operation at t = 24 h (**b**) and t = 144 h (**d**)

Figure 9a shows the average temperature changes of the outlet fluid for double-U tube running for 18 h, stopping for 6 h in intermittent operation and finishing with continuous operation for 7 days. The heat transfer rate for the two working condition is determined with Eq. (3) and is plotted in Fig. 9b. Since intermittent operation can realize cyclical recovery of the ground temperature, the water temperature at the outlet remains at the initial stage and is always lower than the continuous operation mode. Although the operation time of the intermittent mode is lower than that of the continuous mode, the extracted energy is 337.33 kw·h, which is higher than the continuous mode (36.44 kw·h) based on Eq. (3). Figure 10 is the temperature changes of the rock-soil at the radial position (r = 0.078 m, r = 1.4 m) and longitudinal direction (Z = −70 m, Z = −30 m). It is found that the temperature variation trend of the rock-soil at the edge of the borehole (r = 0.078 m) is in keeping with the outlet fluid temperature. The temperature will go down rapidly when the GSHP system shutoff, and increases quickly once the system restarts operation. Interestingly, the rock-soil temperature at r = 1.4 m will not decrease with the GSHP system shutoff. The temperature at R = 0.078 m always greater than R = 1.4 m because it continually receives the heat flux from the heat source. This variation trend does not vary with longitudinal depth. The temperature distribution of nearby soil at −40 m during the operation and out of operation time of the first day and the sixth day are shown in Fig. 11. It can be observed on the 1st day of the outage period, the soil temperature along the depth direction around the buried pipes was obviously lower than during the operational period. In the 6th day of intermittent operation, the change became smaller, and the radius of influence on soil the temperature by the buried pipes gradually increased. In addition, because the gradient of temperature rise of the continuous operation mode at the same position is larger than that of the intermittent operation mode as the operation time increases, the ground temperature in continuous operation mode will be unable to recover, resulting in a much lower efficiency than the intermittent operation mode. Under such operation, other auxiliary cooling and heat sources can be adopted to recover the ground temperature at around the buried pipe area to ensure long-term and efficient running of the system.

3 $$P = \int_{0}^{t} {P(t) \cdot } dt = C_{p} \cdot G\int_{0}^{t} {\left( {T_{out} - T_{in} } \right)} \cdot dt$$

where *G* is mass flow rate in kg/s. *C*_*p*_ is specific heat, J/kg/K. *T*_*in*_ is the temperature of water inlet in K. *T*_*out*_ is the temperature of water outlet in K and *P* is total extracted energy in kW·h.

### Analysis on heat transfer characteristics of different side-tube spacing

**Fig. 12:**
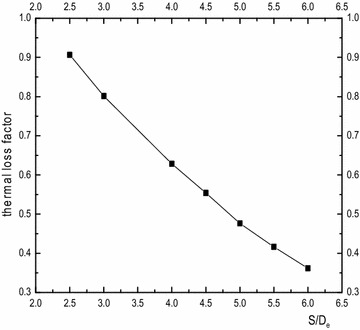
Thermal loss factor of each working condition

**Fig. 13:**
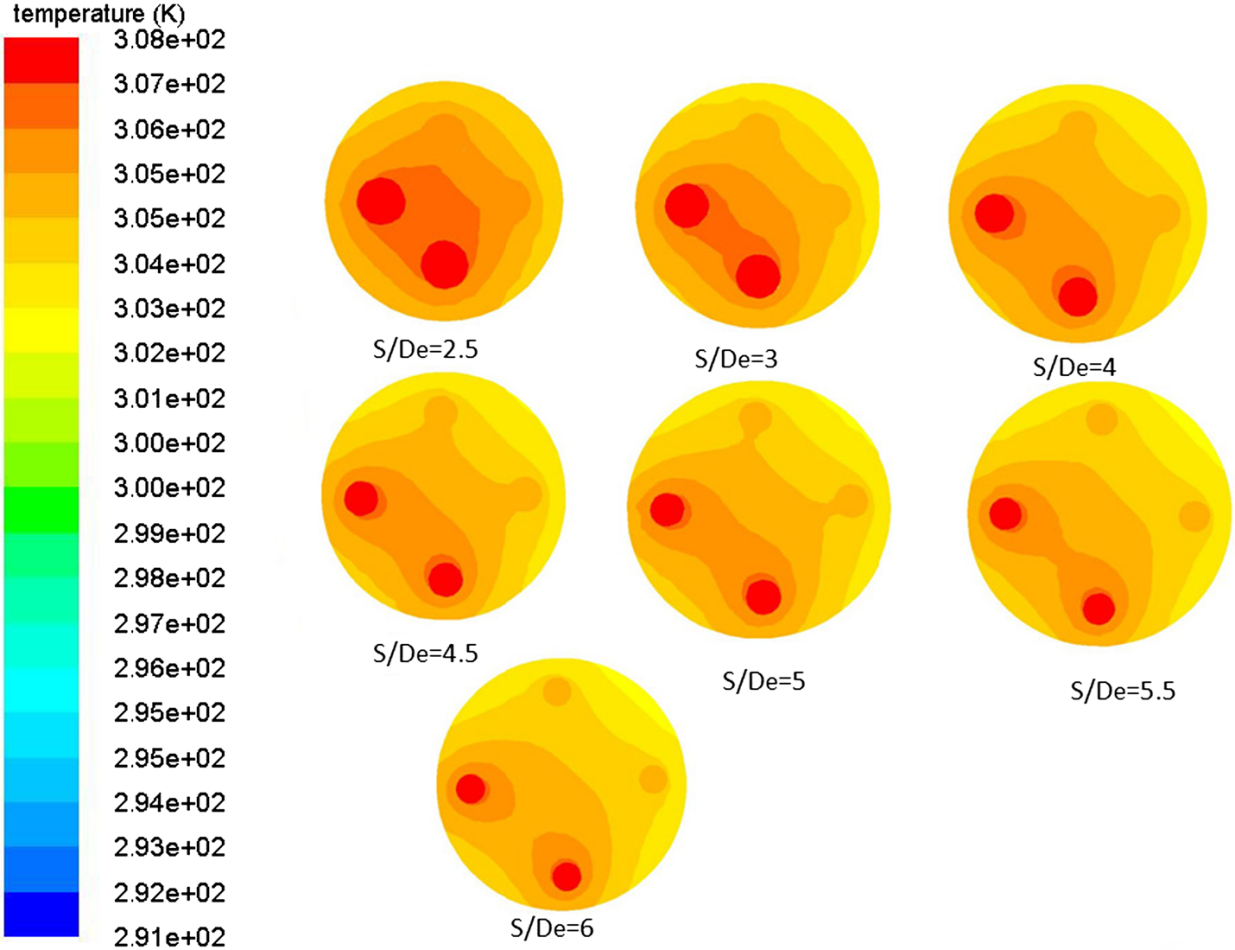
Drill cross section temperature distribution at z = 0

It can be observed above that the interaction effect of the double-U heat exchanger branch pipe is more serious than the single-U model. However, the side-tube spacing has a great impact on the heat transfer of the double-U buried pipe and the selection of proper spacing to achieve economic requirements is worth studying. Under the condition of a side-tube spacing that remains constant, if the tube diameter is bigger, the thermal interference will become more noticeable. Therefore, we use the S/D_e_ value to describe the influence of side-tube spacing to heat transmission effectiveness. Consequently, the branch center distance has reached 2.5, 3, 4, 4.5, 5, 5.5 and 6 De when the heat transfer characteristics of the double-U buried pipe are at a thermal equilibrium state. It is assumed that there is no thermal interference at an infinitely far location, under the circumstance that the temperature difference between inlet and outlet is 4.6 K. Table 4 shows that the double-U heat exchanger heat transfer rate (Q) at seven working conditions, and the ratio between the branch center distance and external diameter of pipe (S/De) is represented by the corner marked i. The comparative calculation between heat transfer rate with an infinitely distant branch interval and heat transfer rate at the working conditions is set up as the heat loss caused by tube pitch. Figure 12 shows the heat loss arising from tube spacing changes. From the figure, it can be observed that when the side-tube spacing increases from S/De = 2.5 to 6, the thermal loss factor gradually decreases from 90.66 to 36.17 % with an increasing inlet/outlet temperature differential. When S/De is greater than 5, the downward gradient of thermal loss starts decreasing slightly. Figure 13 is the drilling surface temperature distribution at z = 0. The results show that the hot fluid inside the U-tube has a great effect on the temperature distribution of the soil around the tube. With the increasing of the tube spacing, the thermal interference gradually weakens. It’s clear that when S/De is greater than five, the backfill soil temperature around the outlet is lower than the outlet temperature, which indicates that the fluid at the outlet will not directly absorb heat from the inlet.

**Table 4:** Comparison of heat transfer rate for seven working condition double-U pipe

S/D_e_	ΔT (K)	Q_i_ (kw)	(Q_∞_ − Q_i_)/Q_i_ (%)
2.5	2.4	5.64	90.66
3	2.54	5.97	80.14
4	2.81	6.6	62.83
4.5	2.944	6.92	55.42
5	3.1	7.28	47.6
5.5	3.23	7.59	41.66

**Fig. 14:**
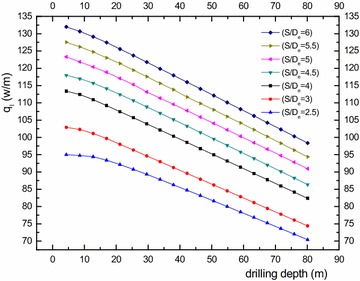
Heat exchange amount of each working condition per unit well depth

Figure 14 is the heat exchange variation of a unit well depth under different spacing conditions. It can be observed from the figure that heat exchange amount rises with the increasing side-tube spacing. From −80 to −20 m, the heat flux of unit well depth presents a linearly increasing trend. However, when S/De < 5, the linearly increasing trend tends to fall from −20 to 0 m. However, when S/De ≥ 5, it almost always presents an increasing linear trend. When S/De increases from 4 to 5, the heat transfer amount at a unit well depth increased by 8.76 %. While increasing from 5 to 6, the heat transfer amount at a unit well depth increases by 8.69 %.

Consequently, under a limited buried pipe area, for a double-U buried pipe system, S/De is suggested to be 5 (with a drilling diameter of 180 mm).

## Conclusions

Based on a CFD numerical simulation method, we analyzed the influence of a GHEs system of GSHP’s on the temperature of rock-soil under the working conditions of heat removal and Studies were carried out on three aspects of performance, such as outlet fluid temperature variation of the double-U buried pipe under continuous operation, intermittent operation and thermal interference arising from different branch pipe spacing. The main conclusion is as follow:

1. After continuous operation for 6 days, a single-well, single-U and double-U GHEs makes the rock-soil temperature increase by 8.0 and 10.38 K at the borehole. In addition, the increasing trend gradually slows down with increasing time. The impact of the double-U buried pipe on the rock-soil temperature is approximately 13 % higher than that of the single-U buried pipe. The thermal interference generated among side-U tubes of double-U buried pipe is 25.48 % higher than that of the single-U pipe. In addition, the impact increases over time. The influence radius of the double-U heat exchanger is twice that of that of single-U heat exchanger.
2. For intermittent operation mode (running for 18 h and shut down for 6 h), the rock-soil temperature obtained a cyclical recovery, and the extracted energy of intermittent is 36.44 kw·h higher than that of continuous mode, although the running time is less than the continuous mode. It is recommended that other auxiliary cooling and heat sources can be adopted during intermittent operation mode to recover the ground temperature to ensure long-term and efficient operation of the system.
3. The heat transfer rate of double-U heat exchanger at the side-U tube at a center distance of S/De as 2.5, 3, 4, 4.5, 5, 5.5 and 6 were compared. It can be observed that when S/De is greater than 5, heat loss decreasing in a small amount. It is undesirable to achieve zero loss from the perspective of economy. In order for this system to be practical, it is suggested to adopt a center distance of S/D_e_ = 5~6 (with drilling diameter of 180~ 205 mm) for single-well double-U buried pipe system

## Abbreviations

GSHP: ground source heat pump
GHE: geothermal heat exchanger

## References

[CR1] ANSYS Fluent 17.0 (2016). Ansys fluent theory guide.

[CR2] ANSYS I. C. 17.0 (2016). User’s manual.

[CR3] Bhutta MMA, Hayat N, Bashir MH, Khan AR, Ahmad KN, Khan S (2012). CFD applications in various heat exchangers design: a review. Appl Therm Eng.

[CR4] Bouhacina B, Saim R, Oztop HF (2015). Numerical investigation of novel tube design for the geothermal borehole heat exchanger. Appl Therm Eng.

[CR5] Carli MD, Tonon M, Zarrella A, Zecchin R (2010). A computational capacity resistance model (CaRM) for vertical ground-coupled heat exchangers. Renew Energ.

[CR6] Choi JC, Lee SR, Lee DS (2011). Numerical and analytical analysis of vertical ground heat exchangers: intermittent operation in unsaturated soil conditions. Comput Geotech.

[CR7] Dehkordi SE, Schincariol RA (2014). Effect of thermal-hydrogeological and borehole heat exchanger properties on performance and impact of vertical closed-loop geothermal heat pump systems. Hydrogeol J.

[CR8] Hu PF, Yu ZY, Na Z, Fei L, Yuan XD (2013). Performance study of a ground heat exchanger based on the multipole theory heat transfer model. Energ Build.

[CR9] Katsura T, Nagano K, Takeda S (2008). Method calculation of the ground temperature for multiple ground heat exchangers. Appl Therm Eng.

[CR10] Launder BE, Spalding DB (1974). The numerical computation of turbulent flows. Comput Methods Appl Mech Eng.

[CR11] Nam Y, Ooka R, Hwang S (2008). Development of a numerical model to predict heat exchange rates for ground-source heat pump system. Energ Buildings.

[CR12] Shang Y, Li SF, Li HJ (2011). Analysis of geo-temperature recovery under intermittent operation of ground-source heat pump. Energ Buildings.

[CR13] Shen GM, Zhang H (2007). Parametric analysis of thermal interference in vertical U-Tube heat exchangers for GSHP. Acta Energies Solaris Sinica.

[CR14] Shevchuk IV, Jenkins SC, Weigand B, Wolfersdorf JV (2011). Validation and analysis of numerical results for a varying aspect ratio two pass internal cooling channel. ASME J Heat Trans.

[CR15] Sun M, Xie JL, Feng XS, Li YL, Liu YS (2000). Polyethylene pipes for water supply (GB/T13663-2000).

[CR16] Tu JY, Yeoh GH, Liu CQ (2009). Computational fluid dynamic: a practical approach.

[CR17] Zhang D, Feng Y, Rong XY (2009). Testing and analysis on underground heat exchange of ground-source heat pump system in a residential district in Chengdu. Build Sci.

[CR18] Zhao J, Dai CS (2007). Design and application of GSHP system.

